# Use of Magnetic Resonance Imaging to Identify Immune Checkpoint Inhibitor–Induced Inflammatory Arthritis

**DOI:** 10.1001/jamanetworkopen.2020.0032

**Published:** 2020-02-26

**Authors:** Ananta Subedi, Sandra G. Williams, Lawrence Yao, Suresh Maharjan, Julius Strauss, Elad Sharon, Anish Thomas, Andrea B. Apolo, Pravitt Gourh, Sarfaraz A. Hasni, James L. Gulley, Mariana J. Kaplan, James D. Katz, Sarthak Gupta

**Affiliations:** 1 Formerly Office of the Clinical Director, Intramural Research Program, National Institute of Arthritis and Musculoskeletal and Skin Diseases, National Institutes of Health, Bethesda, Maryland; 2 WakeMed Physician Practices, Raleigh, North Carolina; 3 Office of the Clinical Director, Intramural Research Program, National Institute of Arthritis and Musculoskeletal and Skin Diseases, National Institutes of Health, Bethesda, Maryland; 4 Clinical Center, Radiology and Imaging Sciences, National Institutes of Health, Bethesda, Maryland; 5 Department of Internal Medicine, Alameda Health System, Oakland, California; 6 Center for Cancer Research, National Cancer Institute, National Institutes of Health, Bethesda, Maryland; 7 Cancer Therapy Evaluation Program, Division of Cancer Treatment and Diagnosis, National Cancer Institute, Bethesda, Maryland; 8 Intramural Research Program, Systemic Autoimmunity Branch, National Institute of Arthritis and Musculoskeletal and Skin Diseases, National Institutes of Health, Bethesda, Maryland

## Abstract

**Importance:**

Immune checkpoint inhibitors (ICIs) have transformed the treatment paradigm for an ever-increasing number of cancers. However, their use has also led to the emergence of immune-related adverse events, such as ICI-induced inflammatory arthritis. A reproducible, reliable, and accessible modality is needed to assess and distinguish early ICI-induced inflammatory arthritis and help in management. Magnetic resonance imaging (MRI) of joints may be helpful for early diagnosis, guiding therapeutic decision-making, and identifying patients at high risk for erosive disease.

**Objective:**

To assess the role of MRI of joints in patients with ICI-induced inflammatory arthritis.

**Design, Setting, and Participants:**

This retrospective case series included patients enrolled at the National Institutes of Health Clinical Center in Bethesda, Maryland. Patients were evaluated by the rheumatology consultation service between December 27, 2016, and May 28, 2019. A retrospective health record review was performed to determine demographic characteristics, clinical characteristics of inflammatory arthritis and malignant tumors, and imaging findings. Inclusion criteria were patients who were enrolled on various institutional review board–approved protocols of ICIs, developed joint-related symptoms, and had MRI data for at least 1 joint. Data were analyzed from June 1, 2019, to September 1, 2019.

**Exposures:**

Undergoing MRI of at least 1 joint.

**Main Outcomes and Measures:**

All MRIs were reviewed for synovitis, tenosynovitis, bone marrow edema, and soft tissue conditions.

**Results:**

A total of 8 patients (mean [SD] age, 58.8 [5.2] years; 6 women and 2 men) between the ages of 50 and 65 years who were undergoing ICI therapy for a variety of malignant tumors were included in this study. Only 1 patient was receiving combined ICI therapy. The results of 13 separate MRI examinations were reviewed. The most commonly performed MRIs were of the hands and wrists (9 MRIs), followed by knee examinations (3 MRIs). Tenosynovitis and synovitis were frequently seen in the hands and wrists. Bone marrow edema and erosions were also found in 3 patients, suggesting early damage. In larger joints (ie, knees and ankles), joint effusions and synovial thickening were characteristic. Most patients (5 patients) were treated with corticosteroids and had good responses. In patients with high-risk features on MRI imaging (eg, bone marrow edema, erosions), disease-modifying antirheumatic drug therapy was also discussed as a treatment option.

**Conclusions and Relevance:**

These findings suggest that advanced imaging may help to distinguish ICI-induced inflammatory arthritis from other causes of joint pain, aid in identifying patients at increased risk of joint damage, and provide utility in monitoring inflammatory arthritis treatment response in patients receiving ICI therapy.

## Key Points

**Question:**

Can magnetic resonance imaging assist with diagnosis and management of immune checkpoint inhibitor (ICI)–associated arthritis?

**Findings:**

In this case series of 8 patients with ICI-induced inflammatory arthritis, magnetic resonance imaging was used to identify synovitis and tenosynovitis as common features of ICI-induced inflammatory arthritis; erosive joint disease was less common but was also detected.

**Meaning:**

These findings suggest that magnetic resonance imaging may provide utility in distinguishing inflammatory arthritis from other causes of joint pain in patients receiving ICI therapy and identifying patients at increased risk of joint damage.

## Teaser

This case series examines the use of magnetic resonance imaging to identify arthritis associated with use of immune checkpoint inhibitors in patients with cancer.

## Introduction

Immune checkpoint inhibitors (ICIs) first gained US Food and Drug Administration approval in 2011 after ipilimumab was found to be efficacious in metastatic melanoma.^1^ Since the approval of ipilimumab, ICIs have shown survival benefit in an increasing number of cancers. These therapies have demonstrated that the immune system can be effectively harnessed to aid in killing cancerous cells. However, their use has also led to the emergence of immune-related adverse events, such as ICI-induced inflammatory arthritis (ICI-IIA), which has been estimated to occur in approximately 2% of patients with cancer undergoing ICI treatments.^2^ Radiographic examinations are commonly performed as part of clinical evaluation; however, radiographs may not be useful during early phases of inflammatory arthritis (IA)^3^ and may only provide indirect information on synovial inflammation. Furthermore, radiographs have low sensitivity for early inflammatory bone involvement and damage. Indeed, despite evident clinical synovitis, patients who develop ICI-IIA typically only show evidence of degenerative changes on roentgenographs.^4^ Musculoskeletal ultrasonography may overcome some of these limitations, but its efficacy is operator-dependent and subject to significant variability in interpretation. In undifferentiated IA, magnetic resonance imaging (MRI) may detect factors associated with the progression to rheumatoid arthritis (RA).^5^ Magnetic resonance imaging findings of bone marrow edema, the combination of synovitis and erosions, and tenosynovitis are significant risk factors for RA progression.^6^ Additionally, MRI can be used to evaluate early changes in the joints and bones in patients with RA before such changes can be detected by other imaging modalities.^7^ Thus, we hypothesized that MRI may be useful to assess early arthritis related to ICI treatment. Anatomic localization of ICI-IIA with the use of MRI could also help in understanding the pathophysiological processes involved in this poorly characterized entity. We aimed to systematically evaluate MRI features of ICI-IIA in a cohort of patients with cancer who were undergoing other treatments at our center.

## Methods

This is a retrospective case series of patients who had given written informed consent and were enrolled in various other institutional review board–approved protocols at the National Institutes of Health Clinical Center in Bethesda, Maryland. All protocols required assessing the clinical activity and safety of immune checkpoint inhibitors, which forms the basis of the data used for this study. This report follows the reporting guideline for case series.^8^ All participants provided written consent to enroll in the respective institutional review board–approved protocols. Patients were evaluated by the rheumatology consultation service between December 27, 2016, and May 28, 2019. We reviewed the medical records of patients with ICI-IIA and identified patients who had undergone MRI evaluation of at least 1 joint as part of their clinical treatment at the onset of symptoms or presentation to our institute. These MRIs were performed for a variety of clinical indications, including exclusion of soft tissue injury or disease, evaluation for subclinical inflammation in patients whose physical findings of IA had resolved but who continued to experience joint pain, and evaluation for subclinical inflammation in a patient with physical examination findings consistent with chronic synovial thickening (ie, joints were swollen but neither warm nor tender). One MRI was also performed to evaluate a patient for progression of erosive disease. For anonymity, patients are identified as numbers between 1 and 8.

### Analysis

With the exception of patients 3 and 7, all MRI examinations included contrast administration. All radiographs and MRIs were reviewed by a musculoskeletal (MSK) radiologist (L.Y.). The MRI assessments of IA included fat-suppressed T-weighted (or short tau inversion recovery sequences) and T1-weighted imaging, and intravenous contrast-enhanced, fat suppressed T1-weighted sequences. Magnetic resonance images were evaluated for the presence of erosion, marrow edema, arthrosis, effusion, synovitis, and tenosynovitis.^9^ Mean, median, and SD calculations were performed. Data were analyzed from June 1, 2019, to September 1, 2019.

## Results

We identified 8 patients (6 women and 2 men), between the ages of 50 and 65 years with mean (SD) age of 58.8 (5.2) years, who underwent MRI of the hands, wrists, knees, or ankles to evaluate MSK concerns that developed after the use of various ICIs (Table). The underlying cancer diagnoses among patients were variable. Joint symptoms started a median (range) duration of 10 (1-44) weeks after initiation of ICI treatment. All patients were undergoing ICI therapy at the time of initial evaluation by the rheumatology consultation service, with the exceptions of patient 4, who had stopped ICI therapy 6 months prior to evaluation, and patient 7, who had stopped ICI therapy 6 weeks prior to evaluation. Patients 3 and 8 were receiving ICI therapy at the time of imaging. The other patients had received their last dose of ICI therapy between 4 weeks and 6 months prior to imaging. On clinical evaluation by a rheumatology consultant, patients had minimal to moderate synovitis or tenosynovitis, and the joint concerns were determined to be clinically secondary to inflammatory disease rather than degenerative disease. Six patients had polyarticular symmetric arthritis, with predominant involvement of small joints, particularly of the wrist. Two patients (patients 6 and 7) had an oligoarticular pattern of joint involvement that affected a large joint.

**Table.** Characteristics and MRI Findings of Patients With Immune Checkpoint Inhibitor Induced Inflammatory Arthritis

Patient No./Sex	Primary Tumor	ICI	ICI Type	Preexisting Joint Disease	Onset of Symptoms, wk^a^	Time From Symptom Onset to MRI, wk	Autoantibody Test Results^b^	Acute Phase Reactants^c^	Findings			Antitumor Response	IA Management
									MSK Examination	Radiographic	MRI		
1/M	Kaposi sarcoma	Pembrolizumab	Anti–PD-1	Tendinopathy (shoulder)	2	18	Negative for RF, CCP, ENA, and ANA	CRP = 13.3 mg/L; ESR = 51 mm/h	Dactylitis, limited passive to active ROM of shoulders, and synovitis in wrists, MCPs, and PIPs	Shoulders: AC joint OA; knee: small effusion; wrists, hands, and feet: unremarkable	Hand: tenosynovitis of bilateral flexor tendons (first though fourth)	Complete	ICI therapy discontinued and high-dose corticosteroids (1 mg/kg) administered with slow taper
2/M	Thyroid cancer	Pembrolizumab	Anti–PD-1	Tendinopathy (shoulder), metastatic disease (pelvis)	4	1	CCP >125; RF = 84; negative for ANA and ENA	CRP = 270 mg/L; ESR = 127 mm/h	Shoulder IA (BL), left biceps tendinitis, first extensor compartment tenosynovitis, effusion in knees, and synovitis in elbows, wrists, PIPs, and knees;	Wrists and hands: Periarticular osteopenia and erosions; left shoulder: unremarkable; feet: degenerative changes	Hand: synovitis of fourth and fifth MCP joint, marginal erosions, and tenosynovitis of the extensor and flexor compartment; periarticular marginal bone marrow edema in carpals and metacarpals	Progressive disease	ICI therapy held; good response to 20 mg/d for 2 wk followed by taper
3/F	Urothelial carcinoma	Nivolumab	Anti–PD-1	OA (hands and knees)	16	9	Negative for RF, CCP, ENA, and ANA	CRP = 6 mg/L; ESR = 57 mm/h	Bilateral first extensor compartment tenosynovitis and arthralgias of wrists, hands, shoulders, knees, and feet	Wrists and hands: degenerative changes	Hand: tenosynovitis of first and sixth extensor compartments	Stable disease	ICI therapy held briefly and high-dose acetaminophen and occupational therapy administered
4/F	Cervical cancer	Nivolumab	Anti–PD-1	None	4	80	Negative for RF, CCP, ENA, and ANA	CRP = 11.3 mg/L; ESR = 48 mm/h	Elbow contractures, restricted subtalar motion, swan neck deformities, effusion in knees, and synovitis in knees, MCPs, PIPs, and wrists	Wrists and hands: joint deformities and diffuse osteopenia; feet and ankles: periarticular osteopenia and bilateral pes planus	Hand: multifocal osseous erosions involving distal radius, distal ulna, carpal bones, and metacarpal bones, synovitis of intercarpal joints, tenosynovitis involving the flexor and extensor tendons at the wrist	Stable disease	NSAIDs and intraarticular corticosteroids administered
5/F	Colon cancer	Avelumab	Anti–PD-L1	None	28	16	ANA = 4.4 ELISA units; negative for dsDNA, ENA, RF, and CCP	CRP = 10.7 mg/L; ESR = 38 mm/h	Effusion in knees and synovitis in wrists, MCPs, PIPs, and knees	NP	Hand: no erosions, tenosynovitis, or synovitis	Progressive disease	Held ICI therapy, prednisone administered with taper, and ICI shortly thereafter discontinued given disease progression
6/F	Pheochromocytoma	Nivolumab and ipilimumab	Anti–PD-1 and anti–CTLA-4	None	24	2	Negative for RF, CCP, ENA, and ANA	CRP = 127 mg/L; ESR = 100 mm/h	Effusion in knees and synovitis in knees and right wrist	NP	Knee: moderate effusion and diffuse thickening of synovium	Progressive disease	Held ICI therapy and therapeutic arthrocentesis, intraarticular corticosteroids, and prednisone 20 mg administered followed by taper
7/F	Cervical cancer	Bintrafusp alfa	Anti–PD-L1 and TGF-βRII trap	None	44	24	ANA = 2.3 ELISA units; negative for RF and CCP	CRP = 107 mg/L; ESR = 111 mm/h	Effusion and synovitis in left ankle and knees	Bilateral knees: minimal degenerative changes; bilateral ankles: soft-tissue swelling	Ankle: complex joint effusion, thickening of tibiotalar joint synovium, and peroneal tenosynovitis	Complete	ICI stopped approximately 6 wk prior to symptom onset owing to another irAE, prednisone 20 mg administered, and methotrexate added given inability to wean steroids
8/F	Lung cancer	Bintrafusp alfa	Anti–PD-L1 and TGF-βRII trap	Seronegative RA	1	13	ANA = 3 ELISA units; negative for RF, CCP, and ENA	CRP = 8.4 mg/L; ESR = 62 mm/h	AC joint warmth, TTP, first compartment tenosynovitis, and synovitis in wrists, MCPs, and PIPs	Hands and wrists: multifocal erosions; shoulders: unremarkable; knees: subtle narrowing of medial compartment on left side	Hand: erosions at the radial styloid and carpal bones; wrist: synovitis and tenosynovitis of the flexor and extensor tendons	Partial	ICI continued and NSAIDs administered for IA

Abbreviations: AC, acromioclavicular; ANA, antinuclear antibody; BL, bilateral; CCP, cyclic citrullinated peptide; CRP, C-reactive protein; CTLA-4, anti-cytotoxic T-lymphocyte-associated protein-4; dsDNA, double-stranded DNA; ENA, extractable nuclear antigen; ELISA, enzyme-linked immunosorbent assay; ESR, erythrocyte sedimentation rate; F, female; IA, inflammatory arthritis; ICI, immune checkpoint inhibitor; irAE, immune-related adverse event; M, male; MCP, metacarpophalangeal; MRI, magnetic resonance imaging; MSK, musculoskeletal; NP, not performed; NSAID, nonsteroidal anti-inflammatory drug; OA, osteoarthritis; PD-1, programmed cell death-1; PD-L1, programmed cell death ligand 1; PIP, proximal interphalangeal; RA, rheumatoid arthritis; RF, rheumatoid factor; ROM, range of motion; TGF-βRII, transforming growth factor β receptor II; TTP, tenderness to palpation.

SI conversion factor: To convert CRP to nanomoles per liter, multiply by 9.524.

^a^ Indicates the time between initiation of ICI therapy and the onset of MSK joint pain.

^b^ Laboratory thresholds for negative results: RF, less than 15 ELISA units; CCP, less than 20 ELISA units; ANA, less than 1 ELISA units; ENA, less than 20 ELISA units. Inflammatory markers were obtained at the time of initial rheumatologic evaluation.

^c^ Reference ranges for ESR, 0 to 25 mm/h; for CRP, 0 to 4.99 mg/L.

Patient 6 presented with persistent unilateral involvement of the knee and subsequently developed contralateral knee and unilateral wrist inflammatory arthritis. This patient had an acute initial presentation of monoarthritis with elevated inflammatory markers (Table). On evaluation by the rheumatology consultation service, she underwent a knee joint aspiration, with synovial fluid consistent with IA, showing minimal red blood cells, elevated white blood cell count (45 000 cells/μL; to convert to ×10^9^ per liter, multiply by 0.001) with preponderance of neutrophils (90%). No crystals were seen, and synovial fluid cultures were negative for aerobic, anaerobic, mycobacterial and fungal organisms.

Patient 7 presented with inflammatory arthritis of the knees and ankles. Most patients (5 patients) also had prominent tenosynovitis. Consistent with other case series,^10,11^ symmetric polyarticular arthritis was seen more commonly in patients receiving ICI monotherapy (6 of 7 patients who received ICI monotherapy). Acute phase reactants and autoantibodies, including antinuclear, rheumatoid factor, and anti–cyclic citrullinated peptide antibodies were tested in all patients. Three patients (patients 5, 7, and 8) had a positive test results for antinuclear antibodies, and 1 patient (patient 2) had positive results for high-titer rheumatoid factor and anti–cyclic citrullinated peptide autoantibodies. Antinuclear antibody test results for patient 5 were positive for low-titer (1.1 enzyme-linked immunosorbent assay units) prior to starting ICI therapy. We did not have data on pre–ICI initiation autoantibody status for any other patient.

### MRI Findings

**Figure 1.**
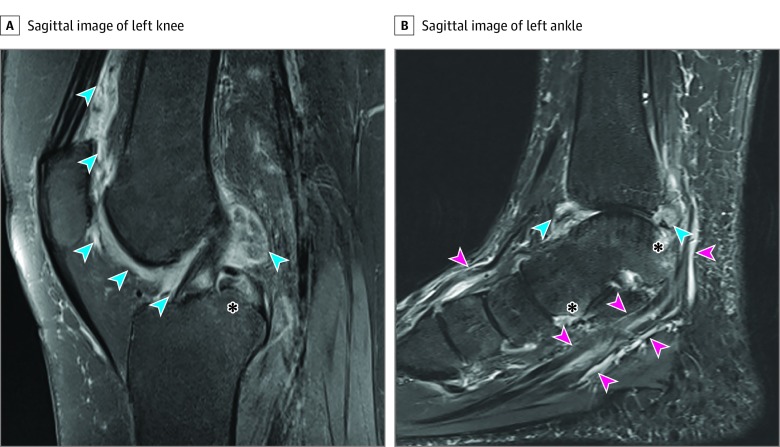
Magnetic Resonance Image of the Knee and Ankle of an Individual With Cervical Cancer and Immune Checkpoint Inhibitor–Induced Inflammatory Arthritis (Patient 7) A, Sagittal fat-suppressed, proton density fast spin-echo image of the left knee depicts extensive, irregular synovial thickening (blue arrows) at the anterior and posterior aspects of the knee and bone marrow edema (asterisk). B, Sagittal image of the left ankle from a short tau inversion recovery sequence demonstrating synovial thickening at the tibiotalar joint (blue arrows), tenosynovitis (pink arrows), and periarticular bone marrow edema (asterisk).

**Figure 2.**
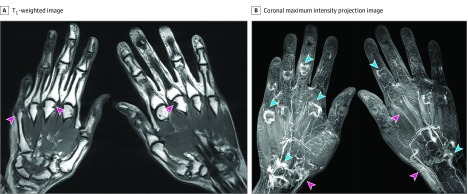
Magnetic Resonance Image of Bilateral Hands in an Individual With Thyroid Cancer With Inhibitor–Induced Inflammatory Arthritis (Patient 2) A, T1-weighted image of both hands reveals multiple marginal osseous erosions (pink arrows). B, Coronal maximum intensity projection image of both hands, generated from gadolinium contrast enhanced T1-weighted 3-dimensional gradient echo images, shows synovial enhancement at metacarpophalangeal, proximal interphalangeal and intercarpal joints (blue arrows), as well as tenosynovitis (pink arrows).

Of 13 MRIS reviewed, the most commonly performed MRIs were of the hands and wrists (9 MRIs), followed by knee examinations (3 MRIs); 1 MRI was performed on a patient’s ankle. Magnetic resonance imaging findings of the small joints (eg, hands) included multicompartment tenosynovitis and bone marrow edema, whereas joint effusion and synovial thickening were observed in large joints (eg, knees and ankles) (Figure 1). Erosions were identified by MRI in 3 patients (patients 2, 4, and 8). Patient 2 did not have a history of IA, but MRI performed 4 weeks after onset of symptoms revealed synovitis, tenosynovitis, bone marrow edema, and erosions (Figure 2). This patient was also found to have elevated levels of anti–cyclic citrullinated peptide autoantibodies, characteristic of RA.

As reported previously,^12^ patient 4 developed arthritis 4 weeks after nivolumab initiation. Despite persistent IA, ICI was continued for 1 year owing to the patient’s apprehension of stopping ICI treatment or receiving systemic therapies for her symptoms, during which period she developed erosive disease with fixed joint deformities. This occurred prior to her clinical trial enrollment at National Institutes of Health. She was followed closely by the rheumatology consultation service for recurrence of IA after she was enrolled on a protocol for bintrafusp alfa, a bifunctional fusion protein composed of the extracellular domain of the transforming growth factor β receptor (TGF-βRII) to function as a TGF-β trap fused to a human IgG1 antibody, blocking programmed cell death ligand 1. Patient 4 had clinical evidence of mild IA but was reluctant to pursue systemic therapy. Follow-up MRI 6 months after initiation of therapy demonstrated persistent synovitis and tenosynovitis but did not demonstrate any new erosions or enlargement of existing erosions when compared with the MRI obtained at the initiation of treatment.

Patient 8 had a history of seronegative RA and positive antinuclear antibody test results. Her arthritis had been in remission without therapy until she developed new onset of joint pain after the initiation of ICI; we do not have records regarding existence of erosions prior to the initiation of ICI therapy.

### Management of ICI-IIA

In our cohort, ICI therapy was held or discontinued in all but 2 patients. The decisions to discontinue ICI therapy were made for several reasons. For patient 1, ICI therapy was discontinued because of MSK complications; this patient had experienced complete response to ICI therapy. For other patients, ICI therapy was discontinued for reasons including disease progression, infectious complications, and other immune-related adverse events. Three patients were treated successfully with nonsteroidal anti-inflammatory drugs or acetaminophen for systemic therapy; 1 of these patients also received intraarticular corticosteroids. The remaining 5 patients experienced complete resolution of their symptoms with various doses of oral prednisone (most patients received 20 mg daily as the initial dose) tapered over a median (range) duration of 5 (2-10) months. Only 1 patient (patient 7) required the addition of methotrexate, a disease modifying antirheumatic drug, which was self-discontinued after 6 months owing to resolution of symptoms. Given erosive disease identified on MRI, patients 2 and 4 were recommended disease modifying antirheumatic drug therapy. However, patient 2 developed serious infectious complications before this was initiated, and patient 4 was very reluctant to use systemic therapy given the perception that it might interfere with her cancer treatment.

## Discussion

This case series found that MRI may be a valuable tool for evaluating ICI-IIA. There is limited evidence regarding the optimal disease assessment tools to distinguish ICI-induced arthralgias from other forms of IA. Physical and radiographic examinations are insensitive for early changes of IA. This could possibly explain the difference in the reported prevalence of arthralgias (from 1% up to 43%)^13^ vs only about 2% for ICI-IIA. Studies detailing the imaging findings of ICI-IIA are sparse. A 2018 study reported that use of radiography in 5 patients showed swelling in soft tissues and narrowing of joint spaces.^14^ In 2 of these patients, MRI demonstrated synovial hyperemia and hyperplasia with adjacent bone marrow edema.^14^ Other previously reported findings include joint effusion, tenosynovitis, and the presence of erosive arthritis in 1 patient.^4,15^ Another option for joint evaluation is MSK ultrasonography, which shows a variety of findings but is dependent on the availability of a trained and experienced operator.^16^

In patients with undifferentiated inflammatory arthritis, specific MRI findings, such as bone marrow edema, both synovitis and erosions, and flexor tenosynovitis, were associated with the development of RA.^6^ In a 2011 study,^5^ bone marrow edema detected on MRI of the metatarsophalangeal and wrist joints was associated with RA progression in 82% of patients with early undifferentiated arthritis. Once rheumatoid arthritis has been diagnosed, MRI is a sensitive test to detect early erosive changes.^9^ These studies all suggest that MRI may be a valuable tool for evaluating ICI-IIA.

In our study, we identified tenosynovitis and synovitis on MRI as common and early radiological features in ICI-IIA, even in patients with minimal symptoms. A subset of 6 patients presented symptoms associated with more aggressive forms IA, including bone marrow edema (3 patients) and osseous erosions (3 patients). The prevalence of erosive ICI-IIA is unknown, to our knowledge. Studies by Richter et al^2^ and Cappelli et al^13^ did not describe osseous erosions as a feature of ICI-IIA. However, in more recent studies, early erosive lesions have been detected by ultrasonography or MRI in a small subset of patients.^4,15,16^ Our findings suggest that erosions may be fairly common and underreported in the absence of sensitive tests, like MRI. Given these findings, it is possible that erosions can occur soon after the onset of symptoms, especially in patients with serologic test results positive for autoantibodies or patients who are partially treated and whose erosive ICI-IIA may represent a much more rapid, aggressive process than is seen in patients with RA.

### Limitations

This study has some limitations. Immune checkpoint inhibitor–induced inflammatory arthritis remains a rare and underrecognized entity that has only recently been described, and our small retrospective case series may limit the generalizability of the study. Also, owing to the heterogeneity of our patient population with respect to the types of cancer and the types of ICIs used, the associations of these variables were difficult to assess. Some patients were already using steroids before undergoing MRI evaluation, and this may have diminished the inflammatory changes on MRI in some of the patients, such as patients 1 and 5. While we did see inflammatory changes on evaluations of 2 patients (patients 3 and 7) who were not administered contrast owing to clinical contraindications, this may also have limited the extent of our findings. Patients in our cohort with autoantibodies offer an insight into the pathogenesis and evolution of this entity; however, because of the retrospective nature of this study, we were unable to assess pretreatment imaging or serologic test results in all the patients, so these are considerations for future prospective studies.

## Conclusions

The findings of this case series suggest that MRI may be useful for early detection of erosive disease, as well as to help identify patients at high risk for erosive disease, and thus guide medical decision-making regarding management of ICI-IIA. This study supports the role of MRI as an important tool in the assessment of ICI-induced articular symptoms. A prospective study of MRI may be fruitful for understanding the pathophysiological processes and long-term clinical implications of this entity. Quantitative measurements through MRI in future studies could potentially help standardize the grading of this adverse event to guide treatment stratification, prevent prolonged exposure to high-dose systemic steroids, and allow early resumption of anticancer therapy.
